# Functional Interactions Between lncRNAs/circRNAs and miRNAs: Insights Into Rheumatoid Arthritis

**DOI:** 10.3389/fimmu.2022.810317

**Published:** 2022-02-07

**Authors:** Juan-Juan Han, Xue-Qiang Wang, Xin-An Zhang

**Affiliations:** ^1^ Department of Sport Rehabilitation, Shanghai University of Sport, Shanghai, China; ^2^ Department of Rehabilitation Medicine, Shanghai Shangti Orthopaedic Hospital, Shanghai, China; ^3^ College of Kinesiology, Shenyang Sport University, Shenyang, China

**Keywords:** noncoding RNA, rheumatoid arthritis, miRNA, circRNA, lncRNA, review

## Abstract

Rheumatoid arthritis (RA) is one of the most common autoimmune diseases that affect synovitis, bone, cartilage, and joint. RA leads to bone and cartilage damage and extra-articular disorders. However, the pathogenesis of RA is still unclear, and the lack of effective early diagnosis and treatment causes severe disability, and ultimately, early death. Accumulating evidence revealed that the regulatory network that includes long non-coding RNAs (lncRNAs)/circular RNAs (circRNAs), micro RNAs (miRNAs), and messenger RNAs (mRNA) plays important roles in regulating the pathological and physiological processes in RA. lncRNAs/circRNAs act as the miRNA sponge and competitively bind to miRNA to regulate the expression mRNA in synovial tissue, FLS, and PBMC, participate in the regulation of proliferation, apoptosis, invasion, and inflammatory response. Thereby providing new strategies for its diagnosis and treatment. In this review, we comprehensively summarized the regulatory mechanisms of lncRNA/circRNA-miRNA-mRNA network and the potential roles of non-coding RNAs as biomarkers and therapeutic targets for the diagnosis and treatment of RA.

## Introduction

Rheumatoid arthritis (RA) is the most common autoimmune diseases with chronic, systemic inflammatory responses; it is characterized by persistent synovitis, bone, cartilage, and joint destruction (1, 2). Symmetrical pain, stiffness, and swelling of one or more joints are the main clinical symptoms of RA; and the joints involved are those in the hands, wrists, feet, and knees (3). The incidence of RA is very high affecting approximately 1% of the world population (4). As the disease progresses, it can lead to bone and cartilage damage and extra-articular disorders, such as cardiovascular disease (5) and organ damage (6); without active clinical treatment, RA can lead to severe disability, and ultimately, early death (2).

RA is a multifactorial and heterogeneous disease; accumulating evidence have documented that genetics is one of its key factors (7). Recently, the influences of environmental factors and gene-environment interactions have been revealed, providing new clues on disease pathogenesis (8). Despite the tremendous research efforts made in the past few years, the precise pathogenesis and etiology are not yet completely elucidated due to the complexity of the disease, resulting in the early diagnosis of RA remains difficult, and existing common serum biomarkers still lack specificity (9, 10). Approximately one third of patients with RA are serologically negative before the onset of severe clinical symptoms (2, 11); thus, many patients miss the best time for early treatment, leading to severe bone and cartilage damage, as well as permanent disability.

Non-coding RNA (ncRNA) is a class of RNA that is unable to encode proteins which mainly include microRNA (miRNA), long non-coding RNA (lncRNA), circular RNA (circRNA), ribosomal RNA (rRNA), transfer RNA (tRNA), small nuclear RNA (snRNA), small nucleolar RNA(snoRNA), small interfering RNA(siRNA), short hairpin RNA(shRNA) and Piwi-interactingRNA (piRNA) (12). In recent years, ncRNA has received much attention (13). ncRNA formerly known as transcriptional noise (14), however, accumulated evidence suggested that ncRNAs could serve as master regulators in a series of biological processes, such as transcription, splicing, and translation; they participate in the development and progression of many diseases, including RA (15, 16). The most commonly studied ncRNAs in RA are miRNAs (17), circRNAs (18), and lncRNAs (19). Numerous studies have shown that miRNAs, lncRNAs, and circRNAs are differentially expressed and participate in regulating the pathological and physiological processes in RA (20, 21). Significantly, new evidence indicated that lncRNAs and circRNAs compete to bind to miRNAs by competitive endogenous RNA (ceRNA) network, and they regulate their target mRNAs in the biological processes of many diseases (22, 23). This crosstalk includes lncRNA/miRNA and circRNA/miRNA, which are essential for the effective regulation of cellular signaling. In this review, we highlight the functional interactions between lncRNAs/circRNAs and miRNAs and describe the crosstalk in the lncRNA/circRNA-miRNA-mRNA axis of RA

## miRNAs and RA

miRNAs belong to a class of short ncRNA molecules that are approximately 22–23 nucleotides in length and are generated endogenously. Mammalian miRNA genes are found in the introns; only approximately 20% miRNAs are found in exons or the combination of exons and introns (24). In general, most miRNAs are named by the combination of miR and a designated number and act as negative regulators; they silence their complementary mRNA expression by cleavage or translation suppression (25). The biosynthesis of miRNAs could be divided into two stages from nucleus to cytoplasm. In the nucleus, miRNA genes located in protein gene introns are transcribed to primary miRNA. Then, they are broken down and converted into pre-miRNA with approximately ~60 nucleotide by Drosha ribonuclease III and diGeorge syndrome critical region 8 (DGCR8); Drosha works by trimming 5’ and 3’ tails (18, 26). After the initial cut, pre-miRNA will translocate to the cytoplasm with the help of the membrane protein exportin 5. In the cytoplasm, miRNA-miRNA duplexes are formed by a Dicer endoribonuclease III known as an endonuclease, which dissociates the secondary structure. After the second initial cut, mature miRNA is formed. Subsequently, one of the miRNA duplexes combine with argonaute protein and transform into RNA-induced silencing complexes (RISCs), and the other is commonly wasted. Eventually, the mature RISC inhibits the translation and expression of target mRNA genes, resulting in the degradation of the message (24, 27).

The biological role of miRNA has been studied extensively for nearly 30 years. Research has shown that miRNAs target a third of all human genes that target mRNA genes and some genes that target DNA (25). In most instances, miRNAs act as inhibitory regulator at the post-transcriptional level by repressing the expression and translation of target mRNA genes, but there are instances when they accelerate the expression level of target genes (28). The miRNAs are highly tissue-specific and differentially expressed in different tissues, and these traits are related to the physiological development and pathological process of a variety of diseases, including cancer (29), stroke (30), heart disease (31), musculoskeletal disease (32), and autoimmune disease (33, 34).

Studies confirmed that miRNAs in synovial tissue, synovial fluid, and blood of patients with RA showed significantly abnormal expression compared with those of healthy individuals (7) (**Table 1**). Synovial tissue is an important part of the knee joint, which mostly includes synovial macrophages and fibroblast-like synoviocytes (FLS) (95); synoviocyte proliferation, invasion, and migration are essential for the RA pathology (96). Among synovial and FLS miRNAs, miR-21 (39), miR-26a-5p (41), miR-126 (50), miR-135a (51), miR-138 (54), miR-143 (56), miR-145 (56), miR-155 (58), and miR-421 (63) are overexpressed, whereas miR-19a (37), miR-20a (38), miR-22 (40), miR-27a (42), miR-29a (44), miR-34a (45), miR-137 (53), miR-140-3p (55), miR−152 (57), and miR-495 (68) are down-regulated. The disturbed miRNAs enhance the expression level of proinflammatory cytokine (IL-6, IL-8, TNF-α, and IL-1β) and enzymes that erode the bone matrix (MMP-1 and MMP-3) by affecting Wnt (97, 98), NF-κB (81, 99), JAK/STAT (48, 100), and TLR (101, 102) pathways. Significantly, the disturbed synovial fibroblast-derived exosomal miRNAs were discovered in recent years. Liu et al. demonstrated that the expression level of miRNA miR-106b was significantly increased in synovial fluid-derived exosomes of RA, and it could target the pyruvate dehydrogenase kinase 4 (PDK4) gene; it could attenuate RA progression by regulating chondrocyte proliferation and migration (47). Furthermore, research found that RA synovial fibroblast (RASF)-exosomal miR-146a, miR-155, miR-323a, and miR-1307 are also involved in inducing local inflammation and attenuating octeoclastogenesis in RA (103).

**Table 1:** The aberrantly expressed miRNAs in RA.

miRNA	Express	Target gene(s)	Tissue/cell source	Model	Species	Functions	Reference
**Synovial tissues**							
miR-10a-5p	Down	TBX5	Synoviocyte	Cell model	–	Proliferation, apoptosis	(35)
miR-17	Down	TRAF2	Synovial tissue, SF, serum	Cell model	Human	Inflammation	(36)
miR-19a	Down	MMP13	Synovial tissues, FLS	Cell model	Human	Proliferation, invasion	(37)
miR-20a	Down	TXNIP	FLS	Cell model	Rat	Inflammation	(38)
miR-21	Up	NF-κB pathway	FLS	Cell model	Human	Proliferation	(39)
miR-22	Down	sirt1	Synovial tissues	Cell model	Human	Proliferation, inflammation	(40)
miR-26a-5p	Up	Smad 1	Synovial tissue	–	–	Invasive	(41)
miR-27a	Down	FSTL1	Synovial tissues FLS, serum	Cell model	Human	Migration, invasion	(42)
miR-27a-3p	Down	TLR5	RASF	Cell model	Human	Apoptosis, inflammation	(43)
miR-29a	Down	STAT3	Synovial tissues, FLS, serum	Cell model	Human	Inflammation, apoptosis	(44)
miR-34a-5p	Down	XBP1	FLS	Cell model	Human	Proliferation	(45)
miR−34a−3p	Down	MDM4	FLS	Cell model	Human	Proliferation, inflammation	(46)
miR-106b	Up	PDK4	SFB-exosomal	Cell model Mouse model	Human Mouse	Proliferation, migration	(47)
miR-124	Down	MARCKS	FLS	Cell model Mouse model	Human Mouse	Proliferation, inflammation	(48)
miR-125	Down	PARP2	Synovial tissues	Rat model	Rat	Inflammation	(49)
miR-126	Up	PIK3R2	Synovial tissues	Cell model	Human	Proliferation, apoptosis	(50)
miR-135a	Up	PIK3R2	Synovial tissues	Cell model	Human	Apoptosis, migration, proliferation	(51)
miR-137	Down	LSD1	Synovial tissue, serum	Cell model Rat model	Human Rat	Inflammation	(52)
	Down	CXCL12	FLS	Rat model	Rat	Proliferation, migration	(53)
miR-138	Up	HDAC4	Synovial tissue, serum	Cell model	Human	Inflammation	(54)
miR-140-3p	Down	SIRT3	SF	Cell model	Human	Apoptosis	(55)
miR-143	Up	IGFBP5	FLS	Cell model	Human	Improve RA-FLS sensitivity	(56)
miR-145	Up	SEMA3A	FLS	Cell model	Human	Improve RA-FLS sensitivity	(56)
miR−152	Down	ADAM10	Synovial tissue, serum, FLS	–	–	Proliferation, inflammation	(57)
miR-155	Up	IKBKE	FLS, PBMC	Cell model	Human	Inflammation	(58)
miR-192	Down	CAV1	Synovial tissue, FLS	Cell model	Human	Proliferation, apoptosis	(59)
miR-193a-3p	Up	IGFBP5	Synovial tissues	Cell model	Human	Proliferation, apoptosis	(60)
miR-221-3p	Up	JAK3	Synovial tissues, synovial fluid	Cell model	Human	Inflammation	(61)
miR-365	Down	IGF-1	Synovial tissues	Mouse model	Mouse	Apoptosis	(62)
miR-421	Up	SPRY1	Synovial tissues, FLS	Mouse model	Human Mouse	Inflammation	(63)
miR-424	Up	DICER1	RASF	Cell model	Human	Apoptosis, proliferation	(64)
miR-431-5p	Down	XIAP	Synovial tissues, FLS	Cell model	Human	Proliferation, apoptosis	(65)
miR-449a	Down	HMGB1	Synovial tissues	Cell model	Human	Inflammation, proliferation	(66)
miR-483-3p	Up	IGF-1	Synovial tissues, FLS	Cell model	Human	Apoptosis, proliferation	(67)
miR-495	Down	β-catenin	Synovial tissues, FLS	–	–	Proliferation, inflammation	(68)
miR-522	Up	SOCS3	SF	Cell model	Human	Inflammation	(69)
miR-3926	Down	TLR5	RASF, synovial tissues	Cell model	Human	Proliferation, inflammation	(70)
miR-6089	Down	CCR4	Synovial tissues, FLS	–	–	Proliferation, apoptosis	(71)
**Synovial fluid**							
miR-574-5p	Up	TLR7/8	Synovial fluid sEV	Cell model	Human	Bone resorption	(72)
miR-146a	Up	FAF1	CD4^+^ T cells of synovial fluid	Cell model	Human	T cell apoptosis	(73)
miR-let7a	Down	HMGA2	synovial fluid macrophages	Mouse model	Human Mouse	Macrophage activation	(74)
**Blood/serum**							
miR-16	Up	RORγt/FoxP3	PBMC/serum	–	–	Th17/Treg imbalance	(75, 76)
	Down	SOX5	FLS/serum	Cell model	Human	Inflammation, migration	(77, 78)
miR-21	Up	–	Plasma	–	–	Biomarkers	(75)
	Down	STAT3	PBMC	Cell model	Human	T-cell homoeostasis	(79)
miR-124	Down	–	Serum	–	–	Related to MMP-3 levels	(80)
miR-125b	Up	NF-κB pathway	Serum/synovial tissues/FLS	Cell model	Human	Inflammation	(81)
	Down	–	PBMC, plasma	–	–	Biomarker	(82)
miR-126-3P	Up	–	Serum	–	–	Biomarkers	(75, 83)
miR-103a-3p	Up	TP53, AGO2	PB, PBMC	–	–	Prognostic biomarker	(84)
miR-155	Up	PU.1/CCL3	PB B cells/serum/PBMC	Cell model	Human	B-cell activation/inflammation	(85–87)
	Down	–	Serum	–	–	Predictors for disease outcome	(77)
miR-146a-5p	Down	CTGF	Serum	Mouse model	Human Mouse	Inflammation, pannus formation	(88)
	Up	–	Plasma/whole blood	–	–	–	(89)
miR-210	Down	–	Serum	–	–	Independent diagnostic markers	(90)
miR-212-3p	Down	SOX5	Serum, synovial tissues, FLS	Cell model	Human	Proliferation, apoptosis	(91)
miR-301a-3p	Up	PIAS3	PBMC	Cell model	Human	Differentiation, proinflammatory,	(92)
miR-5196	Up	–	Serum	–	–	Biomarker	(93)
let-7a	Down	K-Ras, ERK1/2	Monocytes	Cell model	Human	Inflammation,	(94)

RA-SF, rheumatoid arthritis synovial fibroblast; SF, synovial fibroblasts; FLS, fibroblast−like synoviocytes; sEV, small extracellular vesicles; PBMC, peripheral blood mononuclear cell; PB, peripheral blood; TBX5, T-box transcription factor 5; TRAF2, TNF receptor-associated factor 2; MMP-13, matrix metalloproteinase-13; TXNIP, thioredoxin interacting protein; FSTL1, 1follistatin-like 1; TLR5, toll-like receptor 5; STAT3/PIAS3, transcriptionactivator3; XBP1, x-box binding protein 1; MDM4, mouse double minute homolog 4; PDK4, pyruvate dehydrogenase kinase 4; MARCKS, myristoylated alanine-rich C-kinase substrate; PARP2, poly (ADP-ribose) polymerase2; LSD1, lysine−specific demethylase 1; CXCL12, C-X-C motif chemokine ligand 12; HDAC4, histone deacetylase 4; IGFBP5, insulin-like growth factor binding protein5; JAK3, janus kinase 3; IGF-1, insulin-like growth factor-I; SPRY1, sprouty1; XIAP, X-linked inhibitor of apoptosis; HMGB1, high Mobility Group B1; SOCS3, suppressor of cytokine signaling 3; CCR4, CC chemokine receptor 4; FAF1, fas-associated factor 1; HMGA2, high mobility group AT-hook 2; SOX, 5SRY-related high-morbidity-group (HMG) box 5; CTGF, connective tissue growth factor.

As important diagnostic markers for RA, blood miRNAs have been studied extensively. The greatest number of studies focused on miR-146 (miR-146a and miR-146b) and miR-155 (104). However, their roles in RA are still controversial. miR-146a was described to be up-regulated in peripheral blood (PB) and peripheral blood mononuclear cells (PBMCs) from patients with RA (104–106); it is involved in the production of persistent proinflammatory cytokine and disturbance of the balance of Th17-Tregs (107). However, the expression level of miR-146a-5p in synovial fibroblast of RA patients is significantly down-regulated; miR-146a-5p could decrease inflammatory mediators, inhibit angiogenesis, and delay RA progression (88). Furthermore, the anti-rheumatic drugs, such as TNF inhibitors and methotrexate (MTX), could increase miR-146a-5p expression, suggesting that miR-146a-5p may be a potential novel biomarker for predicting and monitoring therapy outcome (87, 108). The roles of miR-155, miR-125b, miR-16, and miR-21 are also ambiguous. Some authors report that miR-155 and miR-16 are over-expressed in serum and PBMC (76, 87), and others report their down-regulation in serum (77, 78). miR-21 and miR-125b are over-expressed in plasma or serum (75, 81) but lowly expressed in PBMC (79, 82). These findings all suggest the tissue specificity of miRNA expression, and its biological function needs to be further studied. The dysregulation of miRNAs is also found in serum exosomes of patients with RA. Wang et al. found that the expression level of miRNA miR-17 was high in RA-exosomes by microarray analysis and real-time PCR; transforming growth factor beta receptor II (TGFBR II) was the direct target. miR-17 could dispute the homeostasis of Tregs by the participation of TGFBR II in the pathogenesis of RA (109). The abundant miRNAs in RA-exosomes provide a new idea and direction for the pathogenesis of RA, which is worthy of further study.

## lncRNAs and RA

lncRNA is a newly identified RNA transcript with a length of more than 200 nucleotides; it has little or no protein-coding potential (15). There are approximately 92 343 lncRNA genes in humans, which are far more than the protein-coding genes (110), and the number is still increasing (111). There are five main categories classified by the localization between lncRNA and the closest protein-coding gene, as follows: intergenic, sense, antisense, intronic, and bidirectional (104, 112). lncRNA was a by-product of RNA polymerase II transcription and was originally called transcriptional noise of the genome; it was considered to have no biological function (113). However, subsequent studies found that lncRNA could regulate target gene expression at each stage from transcriptional and post-transcriptional to post-translational levels (114), and it is also a crucial regulator of a range of cellular transformation processes, such as apoptosis and intracellular transport (24). Furthermore, as a transcription inhibitor, lncRNA could influence the stability of miRNAs and RNA binding proteins as miRNA sponges, and it is involved in the epigenetic modification of DNA (115).

The functional role of dysregulation lncRNAs in the physiological development and pathological process of tumors (116) and cardiovascular disease (117) have been discovered, and the role of lncRNAs in autoimmune diseases was also revealed gradually, but its role is still unclear (118). Studies have discovered that lncRNAs are involved in regulating the development and differentiation of various immune cells, such as thymus T lymphocytes, macrophages, bone marrow B lymphocytes, and dendritic cells (119). lncRNAs are abnormally expressed in RA-associated immune cells and play a crucial role in the physiological and pathophysiological processes.

In recent years, microarray technology has been widely used in the study of RA regulatory network; increasing evidence shows the aberrant expression of lncRNAs in FLS, PBMS, plasma, and synovial tissues in RA patients (113) (**Table 2**). Zhang et al. found 135 differentially expressed lncRNAs (62 up-regulated and 73 down-regulated) and 103 differentially expressed mRNAs (36 up-regulated genes and 67 down-regulated genes) in three pairs of FLS samples through genome-wide analysis of the expression profiles (120). Luo et al. identified 2,410 up-regulated and 2,635 down-regulated lncRNAs and 1,403 up-regulated and 1,886 down-regulated mRNAs in PBMCs *via* microarrays. GO category and KEGG pathway analyses demonstrated that these differentially expressed transcripts are associated with multiple biological processes and signaling pathway, such as T cell receptor signaling pathway and TNF signaling pathway (124). Qin et al. also found that there are approximately 289 differentially expressed lncRNAs and 468 mRNAs in the plasma (127).

**Table 2:** The role of lncRNAs in gene expression profiles of RA.

lncRNAs Up (n)	lncRNAs Down(n)	mRNAs Up (n)	mRNAs Down(n)	Tissue (n)	Species	lncRNAs	Functions	Reference
62	73	36	67	RA-FLS (n=3) Normal-FLS (n=3)	Human	ENST00000483588, uc004afb.1, ENST00000438399,ENST00000452247	Biomarker for RA diagnosis	(120)
190	131	750	1025	RA-synovial (n=5) Normal-synovial (n=5)	Human	RP11-83J16.1	Proliferation, migration, invasion, inflammation	(121)
349	806	1582	1295	RA-synovial (n=3) Normal-synovial (n=3)	Human	lnc-AL928768.3, lnc-AC091493.1	Biomarkers for RA risk and activity	(122)
683	1,416	331	1,976	RA-PBMC (n=3) Normal- PBMC (n=3)	Human	ENST00000456270	Biomarker for RA diagnosis	(123)
2410	2635	1403	1886	RA-PBMC (n=1) Normal- PBMC (n=1)	Human	–	Biomarker for RA therapy	(124)
231	110	–	–	RA-PBMC (n=3) Normal- PBMC (n=3)	Human	MIR22HG, DSCR9, LINC01189, MAPKAPK5-AS1, ENST00000619282	Biomarker for RA diagnosis Apoptosis, autophagy	(125)
275	218	193	181	RA-PBMC (n=2) Normal- PBMC (n=2)	Human	ENST00000569543 ENST00000420096	Biomarker for RA diagnosis	(126)
169	120	280	188	RA-plasma (n=4) Normal- plasma (n=4)	Human	–	Biomarker for RA diagnosis and therapy	(127)

FLS, Fibroblast−like synoviocytes; PBMC, peripheral blood mononuclear cell.

Many aberrantly expressed lncRNAs are reported by microarray analysis, but only a small number of differentially expressed genes participate in the development and progression of RA (**Table 3**). Research demonstrated that lncRNA LERFS (21), MALAT1 (128), UCA1 (130), GAS5 (154), and MEG3 (132) are down-regulated in FLS, whereas GAPLINC (135), Lnc-IL7R (136), ITSN1-2 (137), PVT1 (138), H19 (145), ZFAS1 (155), and PICSAR (139) are up-regulated. The dysregulation lncRNAs are involved in regulating synovial inflammation and cellular biological behavior of RA FLSs, including proliferation, migration, and invasion. lncRNAs may be promising therapeutic targets or biomarkers. Notably, lncRNA HOTAIR shows obvious tissue specificity in different RA tissues. Zhang et al. revealed that HOTAIR was lowly expressed in chondrocytes compared with normal individuals, and miR-138 was the direct target of HOTAIR. HOTAIR usually acts as protective regulator to delay the progression of RA by inhibiting inflammatory response and inactivating the NF-κB signaling pathway (142). Song and his colleagues found that in the differentiated osteoclasts and synoviocytes, HOTAIR was also down-regulated. It could promote the dissolution of bone and cartilage matrix by regulating MMP-2 and MMP-13 expressions. However, in blood mononuclear cells and serum exosomes, HOTAIR was overexpressed and participated in the migration of active macrophage (141). Furthermore, HOTAIR was also found to be up-regulated in serum. Based on these studies, we found that HOTAIR is easy to obtain and detect in blood and is expressed stably, making it a promising biomarker for RA diagnosis.

**Table 3:** The aberrantly expressed lncRNAs in RA.

lncRNA	Express	Target gene(s)	Related genes	Tissue/cell source	Model	Species	Functions	Reference
**FLS**								
LERFS	Down	–	hnRNP Q, RhoA	FLS	Cell model	Human	Migration, invasion, proliferation	(21)
MALAT1	Down	–	CTNNB1	FLS, PBMC	Cell model	Human	Proliferation, inflammation	(128, 129)
UCA1	Down	–	Wnt6	FLS	Cell model	Human	Potential target	(130)
MEG3	Down	–	NLRC5, DNMT1	FLS	Rat model	Rat	Inflammation	(131)
	Down	miR-141	IL-23, Ki67	FLS	Cell model	Human	Inflammation, proliferation	(132)
	Down	–	STAT3, PI3K/AKT	FLS	Cell model	Human	Proliferation, invasion, apoptosis	(133)
GAS5	Down	miR-222-3p	Sirt1	FLS	Cell model	Human	Proliferation, inflammation, apoptosis	(134)
GAPLINC	Up	miR-382-5p, miR-575	–	FLS	Cell model	Human	Proliferation, invasion, migration, proliferation	(135)
Lnc-IL7R	Up	–	EZH2, PRC2	FLS	Cell model	Human	Proliferation, inflammation	(136)
ITSN1-2	Up	–	NOD2, RIP2	FLS	Cell model	Human	Proliferation, inflammation	(137)
PVT1	Up	–	sirt6	FLS	Rat model	Rat	Proliferation, inflammation, apoptosis	(138)
PICSAR	Up	miR-4701-5p	IL-6, IL-8, MMP-3	FLS, synovial fluid	Cell model	Human	Invasion, inflammation	(139)
ZFAS1	Up	miR-27a	MMP-2, MMP-9	FLS, synovial	Cell model	Human	Migration, invasion	(140)
HOTAIR	Up	–	–	Mononuclear	Cell model	Human	Macrophage migration Bone cartilage dissolution	(141)
	Up	–	MMP-2, MMP-13	Osteoclasts, FLS	Cell model	Human		
	Down	miR-138	IL-1β, TNF-α	Chondrocytes	Mouse model, Cell model	Mouse	Proliferation, inflammation	(142)
H19	Up	–	KDM6A	PBMC	Mouse model, Cell model	Human Mouse	M1 macrophage polarization	(143)
	Up	–	Notch, Hes1	Primary synovial cells	Rat model	Rat	Proliferation, apoptosis	(144)
	Up	miR-103a	DDR-2, IL-15	FLS	Mouse model	Human Mouse	Inflammation	(145)
DILC	Down	–	IL-6	Plasma	Cell model	Human	Apoptosis	(146)
ITSN1-2	Up	–	–	Plasma	–	–	Biomarker for RA diagnosis	(147)
**PBMC**								
lncRNA-p21	Down	–	NF-κB, JUNB	PBMC	Cell model	Human	Increase NF-κB activity	(148)
NEAT1	Up	–	STAT3	PBMC, Th17	Mouse model	Human Mouse	inhibit cell differentiation	(149)
NTT	Up	–	C/EBPβ/NTT/PBOV1	PBMC, monocytes,	Cell model	Human	monocyte/macrophage differentiation	(150)
**Lymphocytes**								
LOC100506036	Up	–	SMPD1, NFAT1	T cell	Cell model	Human	Inflammation	(151)
RMBP	Up	–	DDX5-RORγt	Th17cells	Cell model	Human	Th17-mediated inflammatory	(152)
THRIL	Up	–	–	T cell	Cell model	Human	T cell dysfunction	(153)

FLS, Fibroblast−like synoviocytes; PBMC, peripheral blood mononuclear cell; wnt6, wnt family member 6; NLRC5, nucleotide oligomerization domain-like receptor subfamily C5; DNMT1, DNA methyltransferase 1; IL-6/8/15/23, interleukin 6/8/15/23; STAT3, signal transducer and activator of transcription 3; EZH2, zeste homolog 2; PRC2, polycomb repressive complex 2; NOD2, nucleotide oligomerization domain-2; RIP2, receptor-interacting protein 2; MMP-2/3/9/13, matrix metalloproteinase-2/3/9/13; IL-1β, interleukin 1β; TNF-α, tumor Necrosis Factor Alpha; KDM6A, lysine-specific demethylase 6A; DDR-2, discoidin domain Receptor 2; SMPD1, sphingomyelin phosphodiesterase 1.

PBMC is a key component of host defense response and is readily available. However, there are few studies on lncRNA in PBMC at present. Existing studies demonstrated that NEAT1 (149), HIX003209 (156), and NTT (150) are up-regulated in PBMC, whereas H19 (143) and lincRNA-p21 (148) are down-regulated. Regulation of inflammation is an important mechanism of these aberrantly expressed lncRNAs. Yan et al. found that HIX003209 showed a significantly increased expression in PBMC from patients with RA; it could target miR-6089 directly and promote inflammation by regulating the TLR4/NF-κB pathway in macrophages. Furthermore, similar results were observed in lipopolysaccharide-mediated cell models; the overexpressed HIX003209 could function as a positive regulator of proliferation and activation (156). Spurlock et al. found that lincRNA-p21 was down-regulated and NF-κB activation marker phosphorylated p65 was up-regulated by analyzing blood samples and cell culture models from patients with RA; lincRNA-p21 could inhibit NF-κB activity directly. The regulation of lincRNA-p21 was one of the important mechanisms underlying the action of methotrexate against RA (148). Dysregulation lncRNAs, such as LOC100506036, THRIL, and RMBP, were also observed in lymphocytes and macrophages (157). However, their exact functions and mechanisms are still unclear and need further study.

## circRNAs and RA

circRNAs are novel endogenous noncoding RNAs characterized by a closed circular structure and are approximately 500 ribonucleotides (nts) long (34); they include 1–5 exons without intervening introns (158). circRNAs are very stable because of their circular structure, which help them resist exonucleolytic decay through the cellular exosome ribonuclease complex. Studies have shown that circRNAs have a maximum half-life of 48 h, whereas linear mRNAs have only 4–9 h (159). Therefore, circRNAs are ideal biomarkers. The functions of circRNAs include the following: miRNA and RNA binding proteins (RBP) sponge; RNAP II elongation; and RNA maturation regulation (160). circRNAs are widely expressed in mammals and participate in the regulation of physiological and pathological processes for various diseases, such as cancer and osteoarticular and autoimmune diseases (161). Recently, many circRNA-related signaling pathways have been reported in autoimmune diseases, suggesting that circRNAs may serve as crucial immune regulators and potential biomarkers (118).

More aberrantly expressed circRNAs have been identified by gene microarray technology. A series of studies indicated that circRNAs were differentially expressed in PBMC and FLS (**Table 4**). Ouyang and his colleagues detected the expression of circRNA genes in PBMCs from 30 RA patients *via* quantitative real-time polymerase chain reaction (qRT-PCR). They confirmed that circRNAs circRNA_104871, circRNA_003524, circRNA_101873, and circRNA_103047 were up-regulated and may be promising biomarkers for diagnosis (164). Then, Ouyang et al. found that circRNAs were also differentially expressed in plasma. They clarified that circ_0005008 and circ_0005198 were overexpressed in the plasma of RA patients. Furthermore, circ_0005198 may target miR-4778-3p in RA-FLS (166). Wen et al. constructed a circRNA-miRNA network of differentially expressed genes in PBMC from patients with RA, and it contains 165 differentially expressed circRNAs and 63 differentially expressed miRNAs. After further RT-qPCR validation of the four significantly changed circRNAs (circRNA_0001200, circRNA_0001566, circRNA_0003972, and circRNA_0008360), they found that the expression was consistent with the results of sequencing, and these circRNAs may be promising biomarkers for diagnosis (162). After this study, Wen and his colleges further verified the presence of other circRNAs in PBMCs from patients with RA by high-throughput sequencing. The circ_0003353 and circ_0091685 were up-regulated, whereas circ_0005732, circ_0072428 were down-regulated. Then, the expression of circ_0003353 in fibroblast-like synoviocytes was further investigated for functional phenotype analysis; circ_0003353 was significantly highly expressed, it could promote of FLS inflammatory response and cell apoptosis, but inhibited cell proliferation (163).

**Table 4:** The role of circRNAs in gene expression profiles of RA.

circRNAs Up (n)	circRNAs Down(n)	Tissue (n)	Species	circRNAs	Functions	Reference
109	56	RA-PBMC (n=3) Normal-PBMC (n=3)	Human	circ_0001200,circ_0001566, circ_0003972, circ_0008360	Biomarker for RA diagnosis	(162)
109	56	RA-PBMC (n=3) Normal-PBMC (n=3)	Human	circ_0003353	Promote immunity, inflammation, synovial invasion	(163)
9	3	RA-PBMC (n=5) Normal-PBMC (n=5)	Human	circRNA_104871,circRNA_003524, circRNA_101873, circRNA_103047	Biomarkers for RA diagnosis	(164)
41	30	RA-PBMC (n=4) Normal-PBMC (n=3)	Human	circPTPN22	Biomarkers for RA diagnosis	(165)
10	0	RA- plasma (n=5) Normal-plasma (n=5)	Human	circ_0005008, circ_0005198	Disease activity Biomarkers for RA diagnosis	(166)

PBMC, Peripheral blood mononuclear cell.

Although a series of dysregulation circRNAs were found in RA, their downstream pathways in regulating autoimmunity and inflammation are still poorly revealed. Existing studies have shown that the functions of dysregulation circRNAs are involved in regulating synovial inflammation response and cellular biological behavior of RA FLSs, including proliferation, migration, invasion, and apoptosis (**Table 5**). Cai et al. identified that circ_0088194 was up-regulated in RA FLS; it could act as miR-766-3p sponge and promote the expression of downstream target gene MMP2, thereby facilitating the fibroblast−like synoviocytes’ invasion and migration. It may be a novel and promising target for RA (167). Qu et al. suggested that circ-AFF2 was up-regulated in synovial tissues and FLS of RA; circ-AFF2 could bind to the miRNA miR-650; it enhances the expression level of downstream target 2’,3’-cyclic nucleotide phosphodiesterase (CNP) and promotes fibroblast−like synoviocyte proliferation, inflammatory response, migration, and invasion (168). Another study found that the up-regulated circ-AFF2 was also associate with FLS cell progression and inflammatory response *via* the miR-375/TAB2 axis (169). The circRNA circ-Sirt1 was up-regulated in FLS and MH7A cells; it participates in the inhibition of cell proliferation, promotion of apoptosis, and reduction of inflammation in MH7A by targeting the miR-132-mediated Sirt1 pathway (170). Many differentially expressed circRNAs genes should be further validated *in vivo* and *in vitro* to find possible targets and pathways and to provide a theoretical support for the development of novel RA biomarkers and molecularly targeted therapeutic drugs.

**Table 5:** The aberrantly expressed circRNAs in RA.

lncRNA	Express	Target gene(s)	Related genes	Tissue/cell source	Model	Species	Functions	Reference
**FLS**								
circ_0088194	Up	miR-766-3p	MMP2	FLS	Cell model	Human	Invasion, migration	(167)
circ-AFF2	Up	miR-650	CNP	FLS, synovial	Cell model	Human	Proliferation, inflammation, migration	(168)
	Up	miR-375	TAB2	FLS, blood	Cell model	Human	Cell progression, inflammation	(169)
circ-Sirt1	Up	miR-132	Sirt1 pathway	FLS, MH7A cell	Cell model	Human	Proliferation, apoptosis, inflammation	(170)
circ-PTTG1IP	Up	miR-671-5p	TLR4	FLS, synovial	Cell model	Human	Proliferation, inflammation, migration	(171)
circMAPK9	Up	miR-140-3p	PPM1A	FLS	Cell model	Human	Proliferation, inflammation, migration	(172)
circASH2L	Up	miR-129-5p	HIPK2	FLS	Cell model	Human	Growth, motility, inflammation	(173)
circ_0003353	Up	–	–	FLS	Cell model	Human	Proliferation, migration, biomarker	(163)
circ_0008360	Down	miR-135b-5p	HDAC4	FLS, synovial tissue	Cell model	Human	Proliferation, inflammation, migration	(174)
**PBMC**								
circ_09505	Up	miR-6089	AKT1, IκBα NF-κB	PBMC, macrophages	Cell model Mouse model	Human Mouse	Proliferation, inflammation	(175)
ciRS-7	Up	miR-671	mTOR	PBMC	–	Human	Relation of ciRS-7/miR-7/mTOR	(176)
**Plasma**								
circ_0005198	Up	miR-4778-3p	DAS28	Plasma, FLS	Cell model	Human	Biomarkers for RA diagnosis	(166)

## The Crosstalk of lncRNAs, miRNAs, and mRNAs in RA

Mounting evidence demonstrated that lncRNAs could interact with miRNAs in regulating mRNA expression *via* various post-transcriptional mechanisms (177). Four potential mechanisms were associated with the interactions of lncRNA, miRNA, and mRNA (23, 178), as follows: (1) lncRNAs sponge miRNAs as ceRNAs. ceRNA is a kind of RNAs acting as molecular sponges by competing for miRNA response elements (MREs), it could hinder the expression of other target genes, such as mRNAs, by contending with miRNA (179). In the lncRNA-miRNA-mRNA networks, lncRNAs competitively bind to miRNAs as miRNA sponges, inhibit miRNA expression, and enhance the translation of target mRNA. For example, lncRNA PRNCR1 directly binds to miR-326, thereby functioning as a miR-326 “sponge” to up-regulate the expression level of fascin actin-bundling protein 1(FSCN1) in oral squamous cell carcinoma (180). (2) miRNAs degrade lncRNAs. miRNAs directly target lncRNAs and regulate their stability and abundance, thereby affecting different cell functions in physiological and pathological processes. For example, miRNA miR-9 target lncRNA MALAT1, thereby silencing Ago2 and regulating the stability of MALAT1 in the nucleus of L428 (181). (3) lncRNAs bind to target mRNAs and directly compete with miRNAs. The overexpressed LncRNA BACE1AS combines with mRNA BACE1 to reverse the downward trend by miR-485-5p (182). (4) lncRNAs produce miRNAs. Some lncRNAs could generate miRNAs, thereby regulating the expression of downstream genes. For example, lncRNA H19 generates miR-675, thereby inhibiting the expression of insulin-like growth factor 1 receptor(IGF1R) (183). Recently, the lncRNA-miRNA-mRNA networks were gradually revealed to be involved in rheumatic diseases, such as RA (14) and systemic lupus erythematosus (SLE) (184); they participate in biological and pathological processes of diseases. This finding has become a hot topic, thereby attracting increasing attention especially in RA.

**Figure 1:**
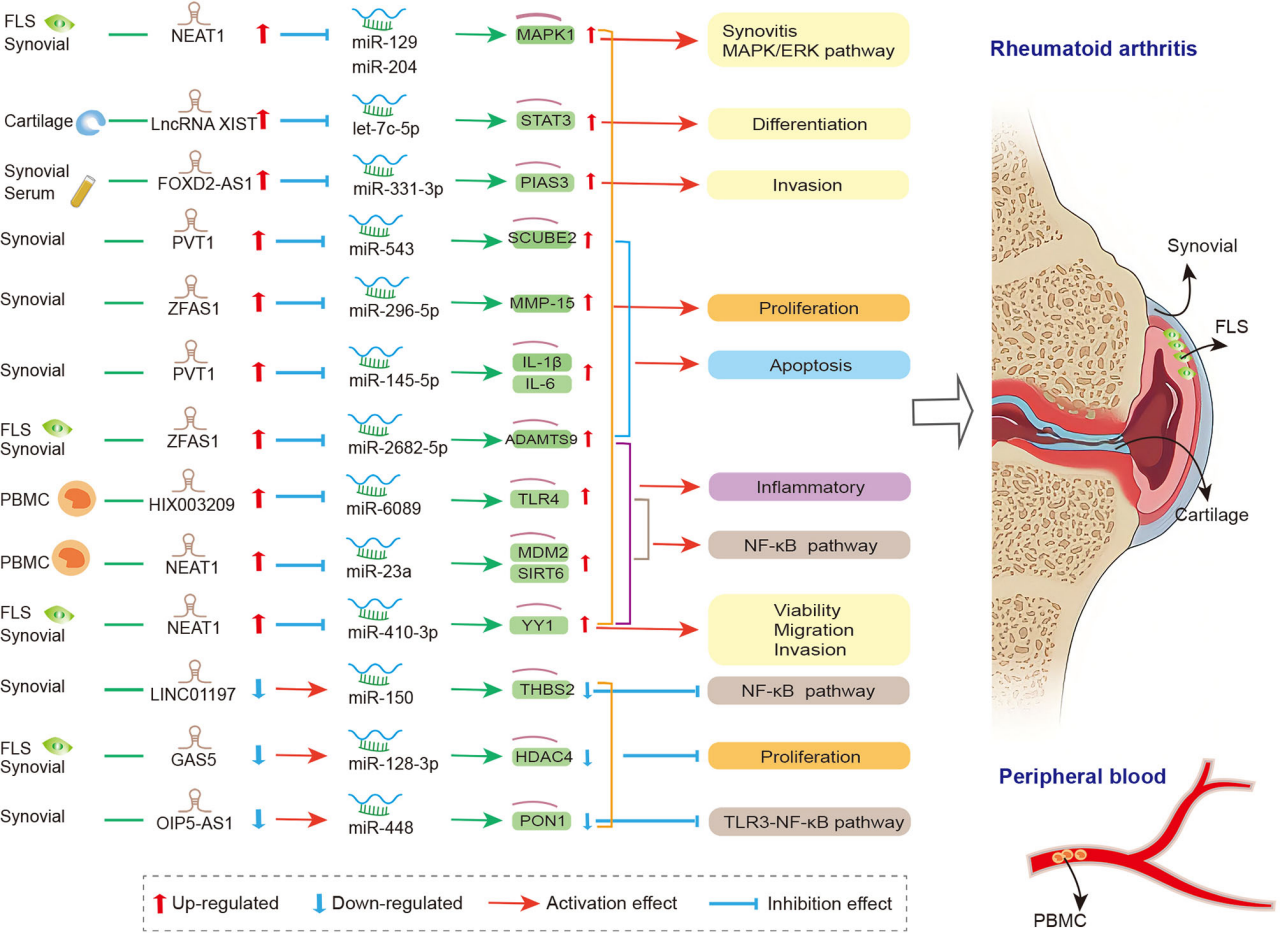
The crosstalk of lncRNA-miRNA-mRNA in RA. FLS, Fibroblast−like synoviocytes; PVT1, plasmacytoma variant translocation 1; SCUBE2, signal peptide-CUB-EGF-like containing protein 2; IL-1β, interleukin-1β; IL-6, interleukin-6; THBS2, thrombospondin-2; GAS5, growth arrest-specific transcript 5; HDAC4, histone deacetylase 4;PIAS3, protein inhibitor of activated STAT3; NEAT1, nuclear paraspeckle assembly transcript 1; MAPK1, mitogen-activated protein kinase 1; YY1, yin yang-1; mdm2, mouse double minute 2; Sirt6, sirtuin 6; PON1, paraoxonase 1; PBMC, peripheral blood mononuclear cell; NEAT1, nuclear paraspeckle assembly transcript 1; MDM2, murine double minute-2; SIRT6, sirtuin 6; MMP-15, matrix metalloproteinase-15; TLR4, toll-like-receptor 4; STAT3, signal transducer and activator of transcription 3.

lncRNAs act as miRNA sponge, and this is the most studied mechanism in RA. lncRNAs competitively bind to miRNAs to regulate the expression level of downstream genes in synovial tissue, FLS, and PBMC. They participate in the regulation of the proliferation, apoptosis, invasion, and inflammatory response of RA-FLSs (**Figure 1**). Zhao et al. detected the expression of lncRNA in the serum and synovial tissues from patients with joint trauma or RA, and they found that the expression of FOXD2-AS1 was significantly increased. FOXD2-AS1 acts as miR-331-3p sponge modulator of downstream target gene STAT3 expression. The overexpressed FOXD2-AS1 increased the proliferation and invasion of fibroblast-like synoviocytes through the miR-331-3p/PIAS3 pathway, suggesting that FOXD2-AS1 may be a promising target for RA treatment (185). Tang et al. reported that in the synovial tissues of RA patients, the expressions of lncRNA PVT1 and miRNA miR-145-5p were negatively correlated. In addition, significantly up-regulated PVT1 and down-regulated miR-145-5p were found in the RA-FLS model (induced by TNF-α). The knockdown of PVT1 could directly target miR-145-5p to inhibit the over-proliferation of RA-FLS and the activation of NF-κB signaling pathway and to regulate the proliferation, apoptosis, and inflammatory response of RA-FLS (186). Wang and his colleagues revealed that the overexpressed PVT1 could directly target miR-543, enhance the expression level of SCUBE2, and promote proliferation and IL-1β secretion while inhibiting the apoptosis rate of FLSs (187). The two abovementioned studies showed that lncRNA PVT1 has two downstream miRNA targets, miR-145-5p and miR-543. Similar to circRNAs, lncRNAs could contain one or more binding sites to miRNAs, thereby serving a sponging function. Studies have clarified that lncRNA NEAT1 could bind to miR-129/miR-204 (188), miR-410-3p (189), and miR-23a (190), thereby regulating cell viability, migration, and inflammation in fibroblast−like synoviocytes from RA. Furthermore, the up-regulated lncRNA ZFAS1 (155, 191), HIX003209 (156), and XIST (192) and down-regulated lncRNA LINC01197 (193), GAS5 (154), and OIP5-AS1 (194) also sponged miRNAs, thereby participating in cell proliferation, differentiation, apoptosis, and inflammation in synovial tissue of RA *via* ZFAS1/miR-2682-5p/ADAMTS9 axis, ZFAS1/miR-296-5p/MMP-15 axis, HIX003209/miR-6089/TLR4 axis, lncRNA XIST/let-7c-5p/STAT3 axis, LINC01197/miR-150/THBS2 axis, GAS5/miR-128-3p/HDAC4 axis, and OIP5-AS1/miR-448/PON1, respectively. The details are presented in **Table S1**.

Accumulating evidence has revealed the crucial role of lncRNA in modulating gene expression through the crosstalk of lncRNA-miRNA-mRNA in the immune and inflammatory pathways of RA. However, a new study found that lncRNA could be regulated by protein coding genes *via* the lncRNA-miRNA-mRNA axis, which was involved in the pathophysiologic process of RA (145). Mu et al. reported that lncRNA H19 was up-regulated, whereas miR-103a was down-regulated in RA-FLS. The expression of H19 could be greatly up-regulated when downstream target discoidin domain receptor 2(DDR-2) was activated, and miR-103a was the direct target of H19. Furthermore, miR-103a acts as a negative regulator that inhibits the expression of downstream genes interleukin 15 (IL-15) and dickkopf 1(DKK1). The study revealed that DDR-2 could exacerbate joint damage and inflammatory response *via* the H19-miR-103a network (145). Another study confirmed that protein coding gene forkhead box M1(FOXM1) was a new transcription regulator of lncRNA (195). Wang et al. indicated that FOXM1 and lncRNA LINC00152 were overexpressed in the FLS of patients with RA. FOXM1 overexpression could promote the expression of LINC00152, thereby acting as a transcription activator. LINC00152 could bind to miR-1270 and negatively regulate its expression. Intriguingly, the study found that the mRNA and protein levels of FOXM1 were positively regulated by LINC00152, and FOXM1 could also bind to LINC00152. Thus, LINC00152 and FOXM1 form a positive feedback loop in RA FLS. In summary, LINC00152 and FOXM1 could competitively bind with miR-1270; FOXM1/LINC00152/miR-1270 is a positive feedback loop involved in regulating the proliferation and apoptosis of RA-FLS (195). These studies revealed a novel molecular mechanism of pathophysiologic process in RA-FLS, thereby providing a new idea and direction for the future study of the pathological mechanism of RA.

## The Crosstalk of circRNAs, miRNAs, and mRNAs in RA

For the past few years, the circRNA-miRNA-mRNA networks were gradually revealed. Studies have shown the presence of multiple miRNA complementary binding sites on circRNAs; circRNAs participate in the regulation of transcriptional and post-transcriptional levels by interacting with miRNA, thereby participating in the biological processes of many diseases (196, 197), such as central nervous system diseases (16), osteoarticular diseases (198), and cancer (199). Two main regulatory mechanisms of circRNA-miRNA-mRNA axis exist, as follows. 1) circRNAs sponge miRNAs. The “sponging” function reveals the regulatory mechanism, i.e., circRNAs may act as mRNA expression regulators by targeting seed sequences, thereby inhibiting the expression of miRNA. circRNA molecules usually contain one or more binding sites to which miRNA binds, thereby serving the sponging function (16). The circ_POLA2/miR-326/GNB1 axis could regulate lung cancer cell stemness and progression. Mechanistically, circ_POLA2 sponging miR-326 functioned as a ceRNA, thereby negatively regulating the expression of miR-326 target GNB1 (200). 2) miRNAs mediate circRNAs. miRNAs target circRNAs, thereby regulating the expression of downstream mRNA genes. miRNA miRNA-1224 could mediate the expression of circRNA circRNA-Filip1l by targeting downstream gene Ubr5, which is involved in the regulation of nociception (201).

**Figure 2:**
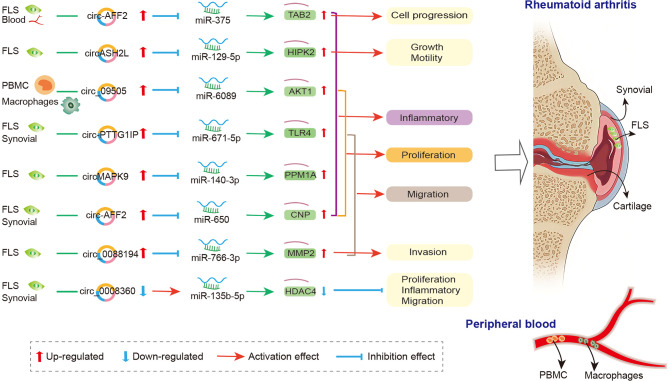
The crosstalk of circRNA-miRNA-mRNA in RA. FLS, Fibroblast−like synoviocytes; PBMC, peripheral blood mononuclear cell; MMP2, matrix metalloproteinase-2; CNP, 2’, 3’-cyclic nucleotide phosphodiesterase; TAB2, binding protein 2; TLR4, toll-like-receptor 4; PPM1A, protein phosphatase 1A; HIPK2, homeodomain-interacting protein kinase 2; HDAC4, histone deacetylase 4.

The crosstalk of circRNAs, miRNAs, and mRNAs was also demonstrated in physiopathological process of RA (174), and the mechanism is that circRNAs act as the miRNA sponge and competitively bind to miRNA, thereby participating in the regulation of downstream genes in FLS, synovial tissue, and PBMC (**Figure 2**). In FLSs from patients with RA, Luo and his colleagues detected the expression levels of circRNA and miRNA *via* qRT-PCR and verified the interaction between them *via* dual-luciferase reporter assay. They indicated that the circMAPK9/miR-140-3p/PPM1A axis was involved in inhibiting inflammatory response, proliferation and migration and accelerating the apoptosis of fibroblast-like synoviocytes. The circRNA circMAPK9 was high expressed and targets miRNA miR-140-3p, and mRNA PPM1A was downstream target gene of miR-140-3p. The knocked down circMAPK9 sponged miR-140-3p and down-regulated PPM1A expression, thereby regulating the biological process of RA-FLSs (172). Hao et al. found that circRNA circ_0008360 was down-regulated in synovial tissue. And then, they used bioinformatics analysis to obtain a preliminary prediction. The results indicated that miR-135b-5p and histone deacetylase 4 (HDAC4) interacted with circ_0008360. They demonstrated that the circ_0008360 sponging miR-135b-5p positively regulated HDAC4 expression, thereby inhibiting the proliferation, migration, and inflammation and facilitating the apoptosis of RA-FLSs (174). Yang et al. found a high expression level of circRNA_09505 in PBMC from patients with RA. The *in vitro* macrophage cell model and *in vivo* collagen-induced arthritis (CIA) mice model indicated that circRNA_09505 could act as miR-6089 sponge through ceRNA mechanism, thereby activating IκBα/NF-κB signaling pathway, promoting miR-6089 direct target AKT1 expression, and exacerbating arthritis and inflammation (175).

Other circRNAs, such as circ_0088194 (167), circ-AFF2 (168), circ-Sirt1 (170), circ-PTTG1IP (171), and circASH2L (173), also acted as miRNA sponge in RA; they regulated FLS proliferation, inflammation, and migration *via* circ_0088194/miR-766-3p/MMP2 axis, circ-AFF2/miR-650/CNP axis, circ-Sirt1/miR-132/Sirt1 pathway, circ-PTTG1IP/miR-671-5p/TLR4 axis, and circASH2L/miR-129-5p/HIPK2 axis, respectively. The details are presented in **Table S2**. The circRNA-miRNA-mRNA networks may have other biological functions in RA diseases besides proliferation, migration, invasion, and inflammatory response. To identify the function of specifically expressed circRNAs and miRNAs, Wen et al. first sequenced circRNAs and miRNAs in PBMCs from three pairs of RA patients and healthy controls. Then, the open source software platform cytoscape was used to build a circRNA-miRNA co-expression network that contained 228 circRNA–miRNA pairs. Further GO and KEGG analyses indicated that the significantly differentially expressed circRNAs were involved in apoptosis, inflammation, autophagy, and oxidative stress (162). This study presents the new idea that circRNAs might be related to the pathogenesis of RA worthy for further study.

## Clinical Implications

RA is the most common autoimmune disease in the world (1). It leads to severe disability and early death (202). Therefore, early detection, diagnosis, and treatment are particularly important (2). Unfortunately, the markers used do not show a high degree of specificity and sensitivity (118). ncRNAs may serve as novel biomarkers because of their characteristics of stable expression in blood and body fluids. In recent years, a growing number of studies confirmed that ncRNAs (lncRNA, circRNA, and miRNA) play key roles in the physiopathological process of RA (34) and may become promising tools for disease diagnosis and prognosis and for prediction of treatment response. Some examples are the ncRNAs in blood (including plasma and serum) and synovial fluid, as follows: lncRNAs MEG3 (203), PICSAR (139); circRNAs circ_0044235 (204), Circ_AFF2 (169), circPTPN22 (165), circ_0005008 (166), and circ_0005198 (166); and miRNAs miR-23b (205), miR-103a-3p (84), and miR-125b (82, 206). Furthermore, several miRNAs could also function as potential biomarkers for RA complication, such as miR-146a-5p and miR-155-5p, which are reported to be possible biomarkers for the development of cardiovascular complications in RA (207). Moreover, the exosome-encapsulated miRNAs, such as miR-548a-3p (208) and miR-150-5p (209), may also be novel and promising targets for RA diagnosis and treatment.

Accumulated evidence demonstrated that the aberrantly expressed ncRNAs offer the opportunity to discover new targeted drugs for patients with RA (107, 161). ncRNA targeting treatment is more selective in RA treatment because of its low susceptibility to infection. Targeting lncRNAs LOC100652951and LOC10506036 modulates T cell inflammation in RA (151). miRNA miR-10a could also act as a regulator of inflammation in RA treatment (210). Furthermore, gene therapy in RA has received much attention in recent years, e.g., RNA interference (RNAi) (211) and antisense oligonucleotides(ASO) (161). RNAi is an intrinsic cellular mechanism that causes mRNA degradation through the interaction of miRNA and small interfering RNA(siRNA) molecules with complementary RNA molecules. Some biologicals of RNAi that target TNF and NF-κB have been used in RA animal models, but the results were not satisfactory (211). However, the treatment of STAT1 siRNA encapsulated by nanoparticles reduced joint deterioration in RA model mice; nanoparticles protected the siRNA from serum degradation (212). ASO is a promising nucleic acid therapy, and the ASO-based drug has been used in many diseases (213). Studies reported that the silencing of miR-223 using lentiviral vectors based on ASO could reduce disease severity of experimental arthritis (214). However, RA-related drugs are lacking. Few studies have investigated the clinical applicability of ncRNAs modulators in autoimmune diseases.

## Conclusions

The studies on ncRNAs, especially the crosstalk of lncRNA/circRNA-miRNA-mRNA in autoimmune disorders, have received much attention. Although a series of published studies have revealed the role of lncRNA/circRNA-miRNA network in regulating inflammation and autoimmunity *via* Wnt3a/β-catenin and TLR/NF-κB signaling pathways in RA, the regulatory mechanism of ncRNAs is still unclear. More in-depth studies are needed to explore the interactions of lncRNA/circRNA-miRNA-mRNA. Elucidating the lncRNA/circRNA-miRNA-mRNA regulatory network and analyzing the interaction mechanism of these fundamental epigenetic regulators in the pathophysiology of RA are still a challenge. With the development of next-generation sequencing and other modern molecular biological techniques, more ncRNA molecular regulatory mechanisms and ncRNA targeted drugs will be uncovered. These would provide new strategies for the clinical diagnosis and targeted treatment for RA.

## Author Contributions

X-AZ and X-QW: conceptualization, project administration, and funding acquisition. J-JH, X-AZ, and X-QW: writing – review and editing. All authors contributed to the article and approved the submitted version.

## Funding

Supported by the Innovative Talents Support Program for Universities of Liaoning Province, No.WR2019024.

## Conflict of Interest

The authors declare that the research was conducted in the absence of any commercial or financial relationships that could be construed as a potential conflict of interest.

## Publisher’s Note

All claims expressed in this article are solely those of the authors and do not necessarily represent those of their affiliated organizations, or those of the publisher, the editors and the reviewers. Any product that may be evaluated in this article, or claim that may be made by its manufacturer, is not guaranteed or endorsed by the publisher.
