# Supplementary material for: Functional Interactions Between lncRNAs/circRNAs and miRNAs: Insights Into Rheumatoid Arthritis
Source: Front Immunol. 2022 Feb 7;13:810317. doi: 10.3389/fimmu.2022.810317 (PMC8858953; doi:10.3389/fimmu.2022.810317)
Supplement: Supplementary file 1 [file DataSheet_1.docx]

Table S1 lncRNA-miRNA-mRNA networks in RA

| **Species** | **Region** | **lncRNA** | **Express** | **miRNA** | **Express** | **Target gene** | **Express** | **Functions** | **Reference** |
| --- | --- | --- | --- | --- | --- | --- | --- | --- | --- |
| Rat | Synovial tissue | PVT1 | Up | miR-543 | Down | SCUBE2 | Up | Promote proliferation, L-1β, inhibit FLS apoptosis | ([Wang et al., 2020a](#_ENREF_156)) |
| Human | Synovial tissue | PVT1 | Up | miR-145-5p | Down | IL‐1β, IL‐6 | Up | Cell proliferation, apoptosis,  NF-κB pathway | ([Tang et al., 2020](#_ENREF_148)) |
| Human, mouse | Synovial tissue | LINC01197 | Down | miR-150 | Up | THBS2 | Down | TLR4/NF-κB inactivation,  ameliorate RA inflammation | ([Zhao et al., 2020](#_ENREF_200)) |
| Human | Synovial tissue,  FLS | GAS5 | Down | miR-128-3p | Up | HDAC4 | Down | Restrain synovial inflammation  AKT/mTOR pathway | ([Peng et al., 2021](#_ENREF_115)) |
| Human | Synovial tissue,  serum | FOXD2-AS1 | Up | miR-331-3p | Down | PIAS3 | Up | Promote FLS proliferation, invasion | ([Zhao et al., 2021](#_ENREF_201)) |
| Human,  Rat | Synovial tissue,  FLS | NEAT1 | Up | miR-129 miR-204 | Down | MAPK1 | Up | FLS proliferation, synovitis  MAPK/ERK pathway | ([Chen et al., 2021a](#_ENREF_13)) |
| Human | Synovial tissue,  FLS | NEAT1 | Up | miR-410-3p | Down | YY1 | Up | Viability, migration, invasion, inflammatory | ([Wang et al., 2020d](#_ENREF_164)) |
| Mice | PBMC | NEAT1 | Up | miR-23a | Down | MDM2, SIRT6 | Up | FLS proliferation, inflammatory | ([Rao et al., 2020](#_ENREF_127)) |
| Rat | Synovial tissue | OIP5-AS1 | Down | miR-448 | Up | PON1 | Down | FLS growth, inflammation，TLR3-NF-κB pathway | ([Qing and Liu, 2020](#_ENREF_121)) |
| Human | Synovial tissue,  FLS | ZFAS1 | Up | miR-2682-5p | Down | ADAMTS9 | Up | FLS proliferation, inflammatory, apoptosis | ([Yang et al., 2020b](#_ENREF_184)) |
| Mice | RA tissues  MH7A cells | ZFAS1 | Up | miR-296-5p | Down | MMP-15 | Up | MH7A cell proliferation, apoptosis | ([Zheng et al., 2021](#_ENREF_202)) |
| Human | PBMC | HIX003209 | Up | miR-6089 | Down | TLR4 | Up | macrophage proliferation, inflammation, IκBα/NF-κB | ([Yan et al., 2019](#_ENREF_179)) |
| Mice | Cartilage  tissues | LncRNA XIST | Up | let-7c-5p | Down | STAT3 | Up | Osteoblast proliferation differentiation | ([Wang et al., 2020g](#_ENREF_169)) |

Abbreviations: FLS=Fibroblast‑like synoviocytes; PVT1=plasmacytoma variant translocation 1; SCUBE2=signal peptide-CUB-EGF-like containing protein 2; IL-1β=interleukin-1β; IL-6=interleukin-6; THBS2=thrombospondin-2; GAS5=growth arrest-specific transcript 5; HDAC4=histone deacetylase 4;PIAS3=protein inhibitor of activated STAT3; NEAT1=nuclear paraspeckle assembly transcript 1; MAPK1=mitogen-activated protein kinase 1; YY1=yin yang-1; mdm2=mouse double minute 2; Sirt6=sirtuin 6; PON1=paraoxonase 1; PBMC=peripheral blood mononuclear cell; NEAT1=nuclear paraspeckle assembly transcript 1; MDM2=murine double minute-2; SIRT6=sirtuin 6; MMP-15=matrix metalloproteinase-15; TLR4=toll-like-receptor 4; STAT3=signal transducer and activator of transcription 3.

Table S2 circRNA-miRNA-mRNA networks in RA

| **Species** | **Region** | **lncRNA** | **Express** | **miRNA** | **Express** | **Target gene** | **Express** | **Functions** | **Reference** |
| --- | --- | --- | --- | --- | --- | --- | --- | --- | --- |
| Human | FLS | circ_0088194 | Up | miR-766-3p | Down | MMP2 | Up | Invasion, migration | ([Cai et al., 2021](#_ENREF_11)) |
| Human | FLS, synovial | circ-AFF2 | Up | miR-650 | Down | CNP | Up | Proliferation, inflammatory, migration | ([Qu et al., 2021](#_ENREF_123)) |
| Human | FLS, blood | circ-AFF2 | Up | miR-375 | Down | TAB2 | Up | Cell progression, inflammatory | ([Zhi et al., 2021](#_ENREF_203)) |
| Human | FLS, synovial | circ-PTTG1IP | Up | miR-671-5p | Down | TLR4 | Up | Proliferation, inflammatory, migration | ([Chen et al., 2021b](#_ENREF_14)) |
| Human | FLS | circMAPK9 | Up | miR-140-3p | Down | PPM1A | Up | Proliferation, inflammatory, migration | ([Luo et al., 2021](#_ENREF_96)) |
| Human | FLS | circASH2L | Up | miR-129-5p | Down | HIPK2 | Up | Growth, motility, inflammation | ([Li et al., 2021](#_ENREF_73)) |
| Human | PBMC, macrophages | circ_09505 | Up | miR-6089 | Down | AKT1 | Up | Proliferation, inflammatory | ([Yang et al., 2020a](#_ENREF_183)) |
| Human | FLS, synovial tissue | circ_0008360 | Down | miR-135b-5p | Up | HDAC4 | Down | Proliferation, inflammatory, migration | ([Hao et al., 2021](#_ENREF_43)) |

Abbreviations: FLS=Fibroblast‑like synoviocytes; PBMC=peripheral blood mononuclear cell; MMP2=matrix metalloproteinase-2; CNP=2', 3'-cyclic nucleotide phosphodiesterase; TAB2=binding protein 2; TLR4=toll-like-receptor 4; PPM1A=protein phosphatase 1A; HIPK2=homeodomain-interacting protein kinase 2; HDAC4=histone deacetylase 4.
